# Prevalence and correlates of passive and active suicidal ideation among students entering graduate courses

**DOI:** 10.1590/1518-8345.6581.3981

**Published:** 2023-09-18

**Authors:** Moisés Kogien, Samira Reschetti Marcon, Camille Francine Modena, Marina Nolli Bittencourt, Larissa de Almeida Rézio, Jesiele Spindler Faria

**Affiliations:** 1 Universidade Federal de Mato Grosso, Cuiabá, MT, Brasil.; 2 Instituto Federal de Mato Grosso, Juína, MT, Brasil.

**Keywords:** Suicidal Ideation, Students, Graduate Education, Risk Factors, University, Mental Health, Ideación Suicida, Estudiantes, Educación de Postgrado, Factores de Riesgo, Universidad, Salud Mental, Ideação Suicida, Estudantes, Educação de Pós-Graduação, Fatores de Risco, Universidade, Saúde Mental

## Abstract

**Objective:**

to identify the prevalence and factors associated with passive and active suicidal ideation throughout life among students entering graduate courses.

**Method:**

an analytical and cross-sectional study with a sample comprised of 321 students entering graduate courses. Multiple descriptive and inferential statistical analyses were performed.

**Results:**

the multivariate analyses indicated that passive and active suicidal ideation were similarly more prevalent among female students belonging to minority sexual orientations who engaged in daily physical activity ≤ 30 minutes/day and were victims of psychological violence in the past 12 months. Alcohol abuse, family relationships not impaired due to the demands imposed by stricto sensu graduate studies and low self-esteem were only associated with passive suicidal ideation. In turn, recent marijuana use in the last 30 days, poor interpersonal relationships with academic peers, and engagement in professional activities concomitantly with the demands imposed by graduate studies were only associated with active suicidal ideation.

**Conclusion:**

high prevalence of lifetime passive and active suicidal ideation was identified among graduate students, and similarities and differences were verified between some associated factors for both outcomes.

## Abstract (es)

**Objetivo:**

identificar la prevalencia y los factores asociados a la ideación suicida pasiva y activa a lo largo de la vida en estudiantes ingresantes de posgrado.

**Método:**

estudio analítico y de diseño transversal, con una muestra compuesta por 321 estudiantes ingresantes de posgrado. Se realizó análisis estadístico descriptivo e inferencial múltiple.

**Resultados:**

los análisis multivariados indican que la ideación suicida pasiva y activa tuvieron una prevalencia mayor y similar en los estudiantes de orientación sexual minoritaria, del sexo femenino, que realizaban actividad física diaria ≤ 30 minutos/día y sufrieron victimización por violencia psicológica en los últimos 12 meses. Los factores abuso de alcohol, las exigencias del posgrado stricto sensu no afectaron las relaciones familiares y baja autoestima se asociaron solo con la ideación suicida pasiva. A su vez, el consumo de marihuana en los últimos 30 días, no tener una buena relación interpersonal con sus pares académicos, ejercer la actividad profesional junto con las exigencias del posgrado se asociaron únicamente con ideación suicida activa.

**Conclusión:**

se identificó una alta prevalencia de ideación suicida pasiva y activa a lo largo de la vida en los estudiantes ingresantes de posgrado y se comprobó que hubo similitudes y diferencias entre algunos de los factores asociados a ambos resultados.

## Abstract (pt)

**Objetivo:**

identificar a prevalência e fatores associados à ideação suicida passiva e ativa ao longo da vida em estudantes ingressantes na pós-graduação.

**Método:**

estudo analítico e de delineamento transversal, amostra composta por 321 estudantes ingressantes da pós-graduação. Realizou-se análise estatística descritiva e inferencial múltipla.

**Resultados:**

análises multivariadas apontaram que ideação suicida passiva e ativa foram similarmente mais prevalentes entre estudantes de orientações sexuais minoritárias, do sexo feminino, que realizavam atividade física diária ≤ 30 minutos/dia e sofreram vitimização por violência psicológica nos últimos 12 meses. O abuso de álcool, o não prejuízo no relacionamento familiar por demandas da pós-graduação stricto sensu e a baixa autoestima foram associadas unicamente com ideação suicida passiva. Por sua vez, o consumo de maconha ocorrido nos últimos 30 dias, a falta de um relacionamento interpessoal satisfatório com seus pares acadêmicos e a prática da atividade profissional em concomitância com as demandas da pósgraduação foram associadas apenas com ideação suicida ativa.

**Conclusão:**

identificou-se alta prevalência de ideação suicida passiva e ativa ao longo da vida entre estudantes ingressantes na pós-graduação e verificou-se a ocorrência de similitudes e diferenças entre alguns fatores associados para ambos os desfechos.

**Highlights:**

**(1)**Advances in the understanding about factors associated with the suicidal ideation spectrum.

**(2)**Differences and similarities between passive and active ideation among graduate students.

**(3)**Recruitment of a homogeneous sample only comprised by students entering stricto sensu graduate courses.

## Introduction

The mental health of *stricto sensu* graduate students has become a topic of increasing concern in recent years, primarily due to a series of evidence pointing to their higher vulnerability to mental distress when compared to students at other educational levels or even the general population ^(^ 1 ^-^ 2 ^)^. 

Graduate education in *stricto sensu* programs takes on different configurations across countries, which imposes unique characteristics for each region of the world. However, some challenges/stressors seem to be universally shared and constitute a complex network of factors intertwined in the personal, social and institutional relationships within training programs ^(^ 3 ^)^. Specific situations experienced in graduate education, such as developing a dissertation or thesis, the pressure for productivity and publication, participation in national and international events, qualification exams and financial difficulties, are commonly reported stressors in the literature. These stressors can result in mental distress in this population ^(^ 3 ^)^. Among the mental distress outcomes to which graduate students are susceptible, those related to suicidal behaviors stand out due to their seriousness and severity ^(^ 4 ^-^ 5 ^)^. 

Suicidal behavior constitutes a *continuum* of multifaceted, multidimensional and complex etiology events that encompass components such as suicidal ideation (passive and active), suicide attempts and suicide itself ^(^ 6 ^)^. Suicidal ideation refers to the phenomenon of having different levels of thoughts about ending one’s own life. It can range from thoughts/wishes about the end of life without a specific plan or method to achieve that goal (passive ideation) to intense thoughts of self-inflicted death with the elaboration of a suicide plan (active ideation) ^(^ 6 ^-^ 7 ^)^. Suicide is a public health problem and one of the leading causes of mortality among young populations ^(^ 8 ^)^, generally found within the context of university training processes across different academic levels ^(^ 8 ^)^. 

Suicidal ideation holds significant clinical and epidemiological relevance because it is a key precursor to suicide attempts and deaths due to suicide, in addition to serving as an important marker for other mental health problems ^(^ 9 ^)^. There is even increasing interest in research studies devoted to clarifying how suicidal ideation can evolve to other manifestations of suicidal behaviors ^(^ 10 ^)^. In the university context, it has been a prevalent health problem among *stricto sensu* graduate students ^(^ 4 ^-^ 5 ^)^. However, there is difficulty comparing these estimates, mainly due to the various time frames under which this outcome has been studied (throughout life, in the last year, or in the last two weeks) ^(^ 2 ^-^ 3 ^)^. In addition to that, studies on mental health among graduate students generally recruit samples of participants from different stages of their graduate studies and there is little distinction regarding the mental health situation between those transitioning into this educational modality and those who have been in it for a longer period of time. This non-distinction makes it challenging to draw precise conclusions about the associative relationships between the graduate educational process and the mental well-being of its students ^(^ 3 ^-^ 4 ^)^. 

Thus, the objective of this study is to identify the prevalence and factors associated with passive and active suicidal ideation throughout life among students entering *stricto sensu* graduate studies. 

## Method

### Study design

This is a study analyzing baseline data from a longitudinal survey on mental illness risk and protective factors among *stricto sensu* graduate students. 

### Study locus and period

The study was conducted from March to April 2021 in a public federal university from Cuiabá, Mato Grosso (MT).

### Population, sample and eligibility criteria

The study population included students entering *stricto sensu* graduate programs (MSc and PhD levels). All students who were beginning their academic training and were regularly enrolled in the first semester of 2021 in any of the 50 *stricto sensu* programs offered across all three *campi* of the university locus of the study were eligible for inclusion. Among the three *campi*, only the main campus offered both MSc (31) and PhD (13) programs, whereas the satellite ones only offered MSc programs (2 and 4, respectively). According to the institution’s Graduate Education Dean’s Office, there were 597 students entering graduate programs during the semester of reference for this study. Among them, 461 were entering MSc programs, whereas 136 were doing so in PhD programs. 

Thus, for the sample estimation, a population size of 597 students entering graduate programs was considered, with a 95% confidence interval, 4% sampling error and an assumed outcome proportion of 50% to ensure the largest possible sample estimate. Considering the aforementioned parameters, the minimum estimated sample size was 299 participants. However, 321 students were recruited to mitigate the potential effects of data losses.

### Study variables and instruments

For the characterization of the passive and active suicidal ideation outcomes, questions from the suicidal behavior assessment module of the Composite International Diagnostic Interview (CIDI) were used, validated for the Brazilian context ^(^ 11 ^)^, and adapted for this study as a self-applied format. 

For the social characterization of the sample, a self-applied questionnaire was developed by the study authors. It contained questions to identify gender, age, self-reported skin color, marital status, sexual orientation, religious practice, current financial concerns and experiences of victimization by interpersonal violence (physical, sexual, and psychological). The occurrence of these experiences in the last year was assessed using adapted questions from a previous study ^(^ 12 ^)^. 

To characterize the academic experiences in graduate studies, an inventory ^(^ 13 ^)^ with a Content Validity Index of 0.93 was used. The instrument assesses the agreement degree (Agree/Disagree) with the experience of potential stressors in *stricto sensu* graduate education, such as the perception regarding the quality of interpersonal relationships with advisors, peers and professors, difficulties with scientific writing, and pressures for productivity. 

In the psychological characterization and assessment of mental health aspects, the following items were evaluated: self-esteem measured by means of the Rosenberg Self-Esteem Scale. This scale consists of 10 items with a 4-point answer format Likert scale and provides a final score from 0 to 30 points ^(^ 14 ^)^. 

Substance use and abuse were assessed through specific questions: alcohol, tobacco and marijuana consumption in the last 30 days (yes/no), as well as alcohol abuse (yes/no) using the CAGE questionnaire (acronym referring to its four questions: Cut down, Annoyed by criticism, Guilty, and Eye-opener) ^(^ 15 ^)^. The practice of physical activity was also evaluated, asking the mean time (in minutes) devoted to performing physical activities in the last 7 days. 

In the characterization of variables related to the COVID-19 pandemic, the perception of fear of COVID-19 was assessed using the COVID-19 Fear Scale ^(^ 16 ^)^ translated and adapted for use in Brazil ^(^ 17 ^)^, including vaccination status, compliance with social distancing/isolation measures, concerns about delays in academic activities due to the pandemic, mental health self-assessment during the pandemic, and identification with any COVID-19 risk group. 

### Data collection

The data were collected by means of an online form whose access link was provided to the participants via the coordination offices of the University’s graduate courses. The eligible students received an invitation letter and the survey link during the first two weeks of data collection. Those who did not respond were sent a reminder invitation during the third and fifth weeks of data collection. The collection phase lasted 45 days. The Informed Consent Form (ICF) was made available online, and the respondents were required to read it and electronically sign it by selecting the dialog box, indicating their understanding of the terms and their agreement to participate in the study. In this stage, each participant was instructed to indicate an email address and/or telephone contact, and this information was used as a marker for duplicate answers/participants. As this was an online study, certain precautions were taken, namely: before starting the questionnaire, the respondents were informed that it would take approximately 15 to 20 minutes to complete. It was also emphasized that they were not obligated to answer any questions that made them uncomfortable, and they had the option to discontinue the study at any time without any consequences.

### Data treatment and analysis

The data of this study were analyzed in the Statistical Package for the Social Sciences (SPSS) software, version 26.0. The descriptive analysis presented the absolute frequencies (AFs) and relative frequencies (RFs) corresponding to each category under study. For the bivariate and multivariate analyses, Poisson regression with robust estimation was used for each of the main study outcomes separately. In the bivariate analysis, the Unadjusted Prevalence Ratio (PR _u_) was reported, with its significance level estimated by means of the Wald test. In order to build the multiple models, all the variables that presented p-values<0.20 in the bivariate analysis were tested, introducing them simultaneously by means of the “backward” technique ^(^ 18 ^)^. The variables that had p-values above 0.05 in the multiple analysis were removed one by one until only the variables with p-values below 0.05 remained in the final model, presenting their adjusted prevalence ratios (PR _a_) and the respective 95% confidence intervals. 

### Ethical aspects

This study follows the national norms on ethics in research with human beings and the Helsinki Resolution; in addition, it was approved by the Health Research Committee of *Universidade Federal de Mato Grosso* (UFMT) under Consubstantiated Opinion No. 4,595,264 and Certificate of Presentation for Ethical Appraisal ( *Certificado de Apresentação de Apreciação* Ética, CAAE): 42807420.0.0000.8124, dated March 2021. 

## Results

The study included 321 students entering *stricto sensu* graduate programs in the first semester of 2021. The sample was characterized by the predominance of female students (72.6%), with a mean age of 32.94 years old (±7.65), students entering MSc courses (86.6%) and from the main *campus* (headquarters) of the university (86.6%). The prevalence values of passive and active suicidal ideation throughout life were 27.4% (n=88) and 16.5% (n=53), respectively. 

Among the social variables which presented p-values<0.20 in the bivariate analysis for at least one of the outcomes of interest in this study, it is noted that female students belonging to minority sexual orientations (homosexual, bisexual, and asexual) and those who reported being victims of psychological violence in the last 12 months were at a higher risk (p<0.05) of both passive and active suicidal ideation. Passive suicidal ideation was also prevalent among students with a median age ≤ 32 years old (p=0.037), whereas active suicidal ideation was prevalent among those who reported not professing any religion (p=0.009) and among those who were victims of some form of sexual violence in the last 12 months (p=0.006), as shown in Table 1. 

**Table 1 -:** Association between passive/active suicidal ideation and social characteristics evaluated in students entering *stricto* *sensu* graduate studies (n=321). Cuiabá, MT, Brazil, 2021

	Passive Suicidal Ideation					Active Suicidal Ideation				
Social characteristics n(%)	Yes	No	PR _u_*	95% CI †	p-value ‡	Yes	No	PR _u_*	95% CI †	p-value ‡
	n(%)				n(%)	n(%)				
Gender										
Female (n=233)	74(31.8%)	159(68.2%)	2.00	1.1-3.34	0.009	45(19.3%)	188(80.7%)	2.21	1.04-4.32	0.038
Male (n=88)	14(15.9%)	74(84.1%)	1.00			8(9.1%)	80(90.9%)	1.00		
Sexual orientation										
Sexual minorities (n=68)	33(48.5%)	35(51.5%)	2.23	1.59-3.13	<0.001	22(32.4%)	46(67.6%)	2.64	1.64-4.25	<0.001
Heterosexual (n=253)	55(21.7%)	198(78.3%)	1.00			31(12.3%)	222(87.7%)	1.00		
Professing some religion										
No (n=72)	25(34.7%)	47(65.3%)	1.37	0.94-2.01	0.104	19(26.4%)	53(73.6%)	1.93	1.18-3.17	0.009
Yes (n=249)	63(25.3%)	186(74.7%)	1.00			34(13.7%)	215(86.3%)	1.00		
Performs some professional activity during the *stricto* *sensu* graduate studies										
No (n=98)	37(37.8%)	61(62.2%)	1.65	1.16-2.34	0.005	25(25.5%)	73(74.5%)	2.03	1.25-3.30	0.004
Yes (n=223)	51(22.9%)	172(77.1%)	1.00			28(12.6%)	195(87.4%)	1.00		
Sexual violence victim										
Yes (n=26)	11(42.3%)	15(57.7%)	1.62	0.99-2.64	0.053	9(34.6%)	17(65.4%)	2.32	1.28-4.20	0.006
No (n=295)	77(26.1%)	218(73.9%)	1.00			44(14.9)	251(85.1%)	1.00		
Psychological violence victim										
Yes (n=147)	54(36.7%)	93(63.3%)	1.88	1.30-2.71	0.001	40(27.2%)	107(72.8%)	3.64	2.03-6.54	<0.001
No (n=174)	34(19.5%)	140(80.5%)	1.00			13(7.5%)	161(92.5%)	1.00		
Age median										
≤32 years old (n=177)	57(32.2%)	120(67.8%)	1.50	1.02-2.18	0.037	35(19.8%)	142(80.2%)	1.58	0.94-2.67	0.086
>33 years old (n=144)	31(21.5%)	113(78.5%)	1.00			18(12.5%)	126(87.5%)	1.00		
Self-reported skin color										
White (n=140)	39(27.9%)	101(72.1%)	1.03	0.72-1.47	0.876	28(20.0%)	112(80.0%)	1.48	0.88-2.37	0.140
Not white (n=181)	49(27.1%)	132(72.9%)	1.00			25(13.8%)	156(86.2%)	1.00		

* PR _u_= Unadjusted Prevalence Ratio;

† 95% CI =95% Confidence Interval;

‡ p-value<0.05 estimated by means of the Wald test

In relation to the academic variables, it is noted that active suicidal ideation was more frequent among those who stated not having a good interpersonal relationship with their university peers (p=0.038). Regarding the pandemic variables, none of them was statistically associated at the p-value<0.05 level with any of the suicidal ideation outcomes (Table 2). 

**Table 2 -:** Association between passive/active suicidal ideation and contextual characteristics (academic and related to the coronavirus pandemic) evaluated in students entering *stricto* *sensu* graduate courses (n=321). Cuiabá, MT, Brazil, 2021

	Passive Suicidal Ideation					Active Suicidal Ideation				
Academic characteristics and those related to the coronavirus pandemic	Yes	No	PR _u_*	95% CI †	p-value ‡	Yes	No	PR _u_*	95% CI †	p-value ‡
	n(%)	n(%)				n(%)	n(%)			
Good relationship with advisor										
Does not agree (n=20)	08(40.0%)	12(60.0%)	1.47	0.83-2.60	0.186	05(25.0)	15(75.0)	1.48	0.66-3.31	0.335
Does not apply (n=22)	04(18.2%)	18(81.8%)	0.67	0.27-1.65	0.382	01(4.5%)	21(95.5)	0.27	0.04-1.86	0.184
Agrees (n=279)	76(27.2%)	203(72.8%)	1.00			47(16.8%)	232(83.2%)			
Good relationship with peers										
Does not agree (n=47)	18(38.3%)	29(61.7%)	1.46	0.96-2.21	0.078	13(27.7%)	34(72.3%)	1.78	1.03-3.07	0.038
Does not apply (n=23)	04(17.4%)	19(82.6%)	0.66	0.26-1.65	0.376	01(4.3%)	22(95.7%)	0.28	0.04-1.94	0.198
Agrees (n=251)	66(26.3%)	185(73.7%)	1.00			39(15.5%)	212(84.5%)	1.00		
Optimism regarding future prospects										
Does not agree (n=67)	23(34.3%)	44(65.7%)	1.34	0.91-1.98	0.142	13(19.4%)	54(80.6%)	1.23	0.70-2.17	0.469
Agrees (n=254)	65(25.6%)	189(74.4%)	1.00			40(15.7%)	214(84.3%)	1.00		
Family relationship impaired by the demands imposed by graduate studies										
Does not agree (n=144)	46(31.9%)	98(68.1%)	1.38	0.94-2.03	0.096	26(18.2%)	117(81.8%)	0.95	0.58-1.57	0.856
Does not apply (n=34)	09(26.5%)	25(73.5%)	1.15	0.61-2.16	0.672	02(5.9%)	32(94.1%)	0.32	0.08-1.30	0.111
Agrees (n=143)	33(23.1%)	110(76.9%)	1.00			25(17.4%)	119(82.6%)	1.00		
Mental health self-assessment during the pandemic										
It got worse (n=252)	75(29.8%)	177(70.2%)	1.58	0.97-2.67	0.088	44(17.5%)	208(82.5%)	1.34	0.69-2.60	0.391
It did not get worse (n=69)	13(18.8%)	56(81.2%)	1.00			9(13.0%)	60(87.0%)	1.00		
Compliance with social distancing measures										
Compliant (n=249)	72(28.9%)	177(71.1%)	1.30	0.81-2.09	0.276	46(18.5%)	203(81.5%)	1.90	0.90-4.02	0.094
Not compliant (n=72)	16(22.2%)	56(77.8%)	1.00			7(9.7%)	65(90.3%)	1.00		

* PR _u_= Unadjusted Prevalence Ratio;

† 95% CI =95% Confidence Interval;

‡ p-value<0.05 estimated by means of the Wald test

Table 3 presents the psychological characteristics and those related to mental health, highlighting that passive and active suicidal ideation were prevalent among students who reported tobacco and marijuana consumption in the last month, had low self-esteem, and did not engage in daily physical activity or did so for ≤ 30 minutes. In turn, an exclusive association with passive suicidal ideation was only observed among students with alcohol abuse behaviors. 

**Table 3 -:** Association between passive/active suicidal ideation and psychological/mental health-related characteristics evaluated in students entering *stricto* *sensu* graduate studies (n=321). Cuiabá, MT, Brazil, 2021

	Passive Suicidal Ideation					Active Suicidal Ideation				
Psychological/ Mental health-related characteristics	Yes	No	PR _u_*	95% CI ^†^	p-value ^‡^	Yes	No	PR _u_*	95% CI †	p-value ‡
	n(%)	n(%)				n(%)	n(%)			
Alcohol consumption in the last month										
Yes (n=201)	54(26.9%)	147(73.1%)	0.95	0.66-1.36	0.775	29(14.4%)	172(85.6%)	0.72	0.44-1.18	0.193
No (n=120)	34(28.3%)	86(71.7%)	1.00			24(20.0%)	96(80.0%)	1.00		
Tobacco consumption in the last month										
Yes (n=43)	17(39.5%)	26(60.5%)	1.55	1.02-2.36	0.042	14(32.6%)	29(67.4%)	2.32	1.38-3.90	0.001
No (n=278)	71(25.5%)	207(74.5%)	1.00			39(14.0%)	239(86.0%)	1.00		
Marijuana consumption in the last month										
Yes (n=26)	13(50.0%)	13(50.0%)	1.97	1.28-3.03	0.002	10(38.5%)	16(61.5%)	2.64	1.51-4.62	0.001
No (n=295)	75(25.4%)	220(74.6%)	1.00			43(14.6%)	252(85.4%)	1.00		
Alcohol abuse										
Yes (n=33)	18(54.5%)	15(45.5%)	2.24	1.55-3.26	<0.001	09(27.3%)	24(72.7%)	1.78	0.96-3.32	0.067
No (n=288)	70(24.3%)	218(75.7%)	1.00			44(15.3%)	244(84.7%)	1.00		
Self-esteem										
Low self-esteem (n=157)	27(50.9%)	26(49.1%)	2.24	1.59-3.16	<0.001	16(30.2%)	37(69.8%)	2.19	1.32-3.63	0.002
High self-esteem (n=164)	61(22.8%)	207 (77.2%)	1.00			37 (13.8%)	231(86.2)	1.00		
Daily time devoted to physical activity										
≤30 minutes/day (n=166)	61(36.7%)	105(63.3%)	2.11	1.42-3.14	<0.001	40(24.1%)	126(75.9%)	2.87	1.60-5.16	<0.001
>30 minutes/day (n=155)	27(17.4%)	128(82.6%)	1.00			13(8.4%)	142(91.6%)	1.00		

* PR _u_= Unadjusted Prevalence Ratio;

† 95% CI =95% Confidence Interval;

‡ p-value<0.05 estimated by means of the Wald test

The results of the multivariate analysis for passive suicidal ideation are presented in Table 4. The model shows that female students (PR _a_=2.44; p<0.001), those belonging to minority sexual orientations (PR _a_=2.36; p<0.001), those who engaged in alcohol abuse (PR _a_=2.01; p<0.001), those who performed physical activity for ≤ 30 minutes a day (PR _a_=1.97; p<0.001), those who stated not having their family relationships impaired by the demands imposed by *stricto sensu* graduate studies (PR _a_=1.91; p<0.001), those who were victims of some form of psychological violence in the last 12 months (PR _a_=1.85; p<0.001), and those had low self-esteem (PR _a_=1.60; p=0.005) were more likely to report passive suicidal ideation throughout life. 

**Table 4 -:** Multivariate analysis with robust estimation of factors associated with passive suicidal ideation among students entering *stricto* *sensu* graduate studies (n=321). Cuiabá, MT, Brazil, 2021

Variable	Category	PR _a_^*^	95% CI †	p-value ‡
Gender	Female	2.44	1.56-3.81	<0.001
	Male	1.00		
Sexual orientation	Sexual minority	2.36	1.76-3.25	<0.001
	Heterosexual	1.00		
Alcohol abuse	Yes	2.01	1.43-2.83	<0.001
	No	1.00		
Daily time devoted to physical activity	≤30 minutes/day	1.97	1.38-2.80	<0.001
	>30 minutes/day	1.00		
Family relationships are impaired due to the demands imposed by *stricto* *sensu* graduate studies	Does not agree	1.91	1.35-2.72	<0.001
	Agrees	1.00		
Psychological violence victim	Yes	1.85	1.32-2.59	<0.001
	No	1.00		
Self-esteem	Low self-esteem	1.60	1.15-2.23	0.005
	High self-esteem	1.00		

* PR _a_= Adjusted Prevalence Ratio;

† 95% CI = 95% Confidence Interval;

‡ p-value<0.05

The model presented in Table 5 shows the results of the multivariate analysis for active suicidal ideation, and it can be observed that four characteristics that acted as risk factors for passive suicidal ideation were also risk factors in the adjusted analysis for active suicidal ideation (minority sexual orientations, female gender, daily time devoted to physical activity ≤ 30 minutes, and being a victim of psychological violence in the last 12 months). In addition to these factors, students who reported marijuana consumption in the last 30 days (PR _a_=2.28; p=0.001) and those who stated not having a good interpersonal relationship with their university peers (PR _a_=2.02; p=0.008) were also at an increased risk for active suicidal ideation. It was also shown that engaging in some professional activity concomitantly with the demands imposed by *stricto sensu* graduate studies acted as a mitigating factor for the prevalence of active suicidal ideation in this sample of students (PR _a_=0.63, p=0.045). 

**Table 5 -:** Multivariate analysis with robust estimation of factors associated with active suicidal ideation among students entering *stricto* *sensu* graduate studies (n=321). Cuiabá, MT, Brazil, 2021

Variable	Category	PR _a_^*^	95% CI ^†^	p-value ^‡^
Sexual orientation	Sexual minority	4.08	2.60-6.42	<0.001
	Heterosexual	1.00		
Psychological violence victim	Yes	3.41	1.94-6.01	<0.001
	No	1.00		
Daily time devoted to physical activity	≤30 minutes/day	3.00	1.76-5.13	<0.001
	>30 minutes/day	1.00		
Gender	Female	2.37	1.36-4.15	0.002
	Male	1.00		
Marijuana consumption in the last 30 days	Yes	2.28	1.41-3.69	0.001
	No	1.00		
Good relationships with peers	Does not agree	2.00	1.31-3.06	0.001
	Agrees	1.00		
Professional activity during the *stricto* *sensu* graduate studies	Yes	0.63	0.40-0.99	0.045
	No	1.00		

* PR _a_= Adjusted Prevalence Ratio;

† 95% CI = 95% Confidence Interval;

‡ p-value<0.05

## Discussion

This study assessed the prevalence of passive and active suicidal ideation throughout life among incoming students entering *stricto sensu* graduate programs at a Brazilian public university and found diverse evidence that these subjects exhibited higher prevalence rates of suicidal ideation than what is typically observed in other population groups. The prevalence values for passive (27.4%) and active (16.5%) suicidal ideation throughout life among graduate studies exceeded the estimations for the general population (9.2% and 3.1%, respectively) ^(^ 19 ^)^. 

Although alarming, the high prevalence values for the suicidal ideation spectrum among graduate students had already been previously pointed out in other studies. This shows that the prevalence of passive suicidal ideation throughout life varies from 25.7% ^(^ 20 ^)^ to 32.2% ^(^ 21 ^)^ and that active suicidal ideation throughout life in Brazilian graduate students is estimated at 19.4% ^(^ 21 ^)^. 

In the final models adopted, the multiple regression analysis for each of the suicidal ideation outcomes evaluated showed that certain characteristics of the sample were significantly associated with both phenomena, supporting the finding of shared similarities among the different suicidal ideation spectra ^(^ 22 ^)^. Minority sexual orientations, female gender, reporting being victims of psychological violence in the last 12 months, and daily mean of physical activity ≤ 30 minutes were shared characteristics among students with a history of both passive and active suicidal ideation. However, the prevalence ratios of these variables were considerably higher among those who reported active ideation in this study. 

The different manifestations of suicidal behaviors, as well as the transition from passive to active suicidal ideation, are frequently associated with the occurrence of triggering circumstances/events that generally carry a significant stress load with them or intensify mental distress/mental health problems ^(^ 6 ^,^ 23 ^)^. These conditions can be related to the findings of this study, as higher prevalence of these outcomes was observed among women, individuals belonging to minority sexual orientations and victims of psychological violence. 

Society has always imposed on women the need to assume multiple and diverse social roles in their everyday life, such as the responsibility of caring for children, older adults household activities. This can be highly exhausting both physically and mentally when they need to balance these demands with personal, professional or academic development activities ^(^ 24 ^-^ 25 ^)^. This myriad of roles that are simultaneously performed and expected can lead to role conflicts and to negative psychological repercussions which, when combined with the time and energy required to manage all these specific requirements, become conducive to the installment of a situation that inevitably leads to mental distress and suffering, ultimately triggering the manifestation of suicidal thoughts ^(^ 24 ^)^. 

In a similar perspective, mainly regarding the cumulative effect of experiencing stressful events throughout their life, the literature has consistently pointed out that individuals belonging to minority sexual orientations are at a higher risk of reporting suicidal ideation when compared to their heterosexual counterparts ^(^ 26 ^-^ 27 ^)^. While trying to navigate the academic context of graduate studies, many students belonging to minority sexual orientations must mobilize adaptive energy to cope not only with the academic stressors but also with stigmatization, discrimination, prejudice, rejection, homophobia, transphobia and other forms of aggression ^(^ 28 ^)^, both within the academic environment and in their personal lives outside university. In addition to that, a meta-analysis aiming to characterize the phenomenon of passive suicidal ideation and compare it with active ideation highlights the relevance of the relationship between minority sexual orientations and this outcome, as it presents the largest effect size among the sociodemographic variables analyzed ^(^ 29 ^)^. 

The associations between being victims of different forms of interpersonal violence and suicidal thoughts in different graduate courses have been strongly reported in the literature ^(^ 30 ^-^ 31 ^)^. However, despite this significant relationship, victimization by interpersonal violence, including psychological violence, does not appear to be a directly associated factor in the etiology of suicidal ideation. Instead, it is a distal factor that exerts impacts on the manifestation of this type of thoughts mediated by more proximal factors caused by victimization, such as depressive and somatic symptoms, and psychoactive substance use ^(^ 27 ^)^. Nevertheless, it is worth noting that being a victim of interpersonal violence can generate severe physical and psychological sequelae that may result in mental distress and in the manifestation of suicidal thoughts ^(^ 31 ^)^. 

Diverse evidence compiled in a systematic review and meta-analysis has pointed out that practicing physical activity is a promising intervention for mitigating suicidal ideation ^(^ 32 ^-^ 33 ^)^. However, it is still uncertain what physical activity types and doses (time) are ideal to achieve this effect ^(^ 30 ^)^. On the other hand, there is greater clarity from previous evidence regarding the detrimental effects of sedentary behavior on mental health ^(^ 34 ^)^. This perspective is in line with the findings of this study, which evidenced that both active and passive suicidal ideation are more prevalent in individuals who do not practice physical activity or do so for brief periods of time (≤ 30 minutes a day). 

It is worth noting that sedentary lifestyles have been associated with increased psychological distress and with a greater sense of hopelessness, which are significant triggers for suicidal behavior ^(^ 35 ^)^. In addition to that, it is important to note the high prevalence of students presenting symptoms that are compatible with depression in this sample, with inertia and sedentary lifestyle as common manifestations of this condition ^(^ 36 ^)^. 

Furthermore, in addition to the shared similarities between passive and active ideation upon entering graduate courses, each of these outcomes presented unique factors associated with their prevalence. This is consistent with the findings of a study conducted with aged Chinese individuals, which found that factors associated with passive ideation, active ideation and suicide attempts differed among each of these outcomes ^(^ 6 ^)^. 

In relation to passive ideation, students with low self-esteem, engaged in alcohol abuse and who did not have their family relationships impaired by the demands imposed by graduate studies presented higher prevalence values of this outcome. As they are not associated with active suicidal ideation, these findings may suggest that these elements are somehow related to the onset of passive suicidal thoughts but may not be important factors in the transition from passive to active ideation ^(^ 18 ^)^, at least in this sample. 

Low self-esteem is a recurring risk factor for suicidal ideation, and it is believed that the recurrence of a pessimistic view of future prospects and a predominant perception of worthlessness can be associated with the occurrence of suicidal thoughts ^(^ 37 ^)^. 

In relation to *stricto sensu* graduate studies, although this is not a consensus in the literature, it is acknowledged that entering this academic level represents a turbulent period and a vulnerability for students ^(^ 38 ^-^ 39 ^)^, mainly regarding their self-esteem. During this transitional period, students are inclined to make social comparisons to evaluate their own worth. However, these comparisons are not always beneficial and can accentuate a series of negative feelings such as worthlessness, low self-esteem and a sense of social alienation ^(^ 40 ^)^. 

The use of psychoactive substances, including alcohol and marijuana consumption/abuse, has been documented as a major public health problem among young adults, including those in university settings, with repercussions not only on academic performance but also on physical and mental health ^(^ 41 ^)^. In a previous study conducted with a similar population and in the same regional context, high prevalence (40.17%) of alcohol abuse was found among graduate students ^(^ 42 ^)^. 

The fact that the students included in this sample who stated that their family relationships were not impaired by the demands imposed by graduate studies had higher prevalence of passive suicidal ideation than those who perceived some negative impact can be explained by the non-evaluation of other factors that may affect family dynamics, which were not considered in this study. In addition to that, it is important to note that, during data collection, inclusion in this teaching modality had taken place less than 45 days before.

The relationship between the quality of family relationships and suicidal behavior has been a recurrent and well-documented topic in the literature. The available evidence has shown that poor or conflictive family dynamics are associated with higher prevalence of suicidal outcomes ^(^ 43 ^-^ 44 ^)^. This is mainly because these relationships become a significant source of distress and do not serve as supportive networks that might assist in mitigating mental distress and encourage seeking help in the case of suicidal thoughts, for example ^(^ 44 ^)^. 

Some variables included in this research were only associated with active suicidal ideation throughout life, namely: marijuana consumption in the last 30 days: disagreeing on having good interpersonal relationships with academic peers: and engaging in some professional activity concomitantly with the demands imposed by graduate studies. This latter variable was characterized as a protective factor in this analysis. Although this association was found, the confirmation that these variables contribute to the transitional process from passive to active ideation, as well as the mechanisms through which this takes place, is still limited, and future studies in this perspective need to be conducted.

Marijuana consumption has been associated with suicidal ideation, particularly its frequent use ^(^ 45 ^-^ 46 ^)^. This consumption can affect psychopathological characteristics related to an increase in suicidal thoughts ^(^ 47 ^-^ 46 ^)^. In addition to that, it is possible that students with a history of active suicidal ideation and more severe suicidal behavior engage in marijuana consumption as a negative coping strategy to alleviate mental distress ^(^ 45 ^-^ 47 ^)^. 

Good quality interpersonal relationships with academic peers are characterized as an important relational factor in the experience of *stricto* *sensu* graduate studies and have the potential to predict better indicators of academic success and even mental health ^(^ 48 ^)^. In a study conducted with students entering PhD programs, interaction with academic peers consistently predicted emotional and experiential outcomes such as a sense of belonging and satisfaction with academic performance ^(^ 48 ^)^. It is deemed important to emphasize that the data were collected during a pandemic period when in-person classes were suspended and only remote teaching and research activities were in effect. All academic social interactions, whether with professors, advisors or peers, occurred exclusively through virtual technologies. 

Of the associated factors found in this study, the only one that was characterized as protective was the “with a job at the beginning of *stricto sensu* training” work status. Despite the potential negative effects of work-related stress on mental health, having a job is a notorious protective factor against suicidal behaviors ^(^ 49 ^-^ 50 ^)^. Regardless of their working conditions, employed individuals who are able to earn a regular income may feel financially secure, which positively impacts their sense of well-being and helps minimize mental distress and suicidal thoughts ^(^ 49 ^-^ 50 ^)^. 

The cross-sectional design precludes establishing causal links between the variables under study. Although a wide range of variables was included in this study, it was not possible to control the analyses for all social, demographic, and academic characteristics of the sample. Combined with the observational nature of the research, this increases the possibility of residual confounding. Finally, we highlight the limitations referring to the sample size, which, although representative of the local population under study, may lead to an increased risk of Type II error in certain multivariate analyses.

It is believed that the results of this study can assist nurses and other mental health professionals in better understanding the different aspects of suicidal ideation and its manifestations among graduate students, in addition to providing valuable insights for clinical decision-making in the prevention of this phenomenon. It is worth emphasizing that early identifying suicidal ideation is an important healthcare measure to prevent suicides. Consequently, the findings of this study may assist in the elaboration of university policies targeted at mental health promotion with a focus on reducing the effects of suicidal thoughts.

## Conclusion

A high percentage of students entering *stricto sensu* graduate studies with a history of passive and/or active suicidal ideation was identified. In the analysis of associated factors, it was verified that graduate students who identified as belonging to sexual minorities and to the female gender had a history of psychological violence in the last 12 months and did not practice physical activity or did so for ≤ 30 minutes a day were at a higher risk for a history of both passive and active suicidal ideation throughout their life. In addition to that, low self-esteem, alcohol abuse, and not having family relationships impaired by the demands imposed by graduate studies were factors exclusively associated with a history of passive suicidal ideation. On the other hand, marijuana use in the last month, not stating not having a good interpersonal relationship with academic peers and engaging in some professional activity during the graduate studies were associated with a history of active suicidal ideation, with this latter being characterized as a protective factor. 
